# Biochemical Properties and Potential Applications of Recombinant Leucine Aminopeptidase from *Bacillus kaustophilus* CCRC 11223

**DOI:** 10.3390/ijms12117609

**Published:** 2011-11-07

**Authors:** Yanfei Shen, Fanghua Wang, Dongming Lan, Yuanyuan Liu, Bo Yang, Yonghua Wang

**Affiliations:** 1 Department of Biotechnology, School of Bioscience & Bioengineering, South China University of Technology, Guangzhou 510006, Guangdong, China; E-Mail: petsyf@126.com; 2 College of Light Industry and Food Sciences, South China University of Technology, Guangzhou 510640, Guangdong, China; E-Mails: sdzbwfh@126.com (F.W.); lyy0537@163.com (Y.L.); 3 School of Chemistry and Chemical Engineering, South China University of Technology, Guangzhou 510640, Guangdong, China; E-Mail: landongming@yahoo.com.cn

**Keywords:** leucine aminopeptidase, biochemical properties, circular dichroism spectroscopy, *Bacillus kaustophilus* CCRC 11223, hydrolysis, anchovy

## Abstract

Experiments were carried out to investigate the effects of various factors on the activity and conformation of recombinant leucine aminopeptidase of *Bacillus kaustophilus* CCRC 11223 (BkLAP) and potential utilization of BkLAP in the hydrolysis of anchovy protein. Optimal temperature and pH of BkLAP were 70 °C and 8.0 in potassium-phosphate buffer, respectively, and the activity was strongly stimulated by Ni^2+^, followed by Mn^2+^ and Co^2+^. Conformational studies via circular dichroism spectroscopy indicated that various factors could influence the secondary structure of BkLAP to different extents and further induce the changes in enzymatic activity. The secondary structure of BkLAP was slightly modified by Ni^2+^ at the concentration of 1×10^−4^ M, however, significant changes on the secondary structures of the enzyme were observed when Hg^2+^ was added to the concentration of 1×10^−4^ M. The potential application of BkLAP was evaluated through combination with the commercial or endogenous enzyme to hydrolysis the anchovy protein. Results showed that combining the BkLAP with other enzymes could significantly increase the degree of hydrolysis and amino acid component of hydrolysate. In this regard, BkLAP is a potential enzyme that can be used in the protein hydrolysate industry.

## 1. Introduction

Every year, industrial processing of feeds destined for human nutrition and animal consumption results in high amounts of agroindustrial residues. These residues are receiving greater attention in terms of quality control and have been classified as agroindustrial by-products [1]. Nowadays, with the increasing awareness about the environment, scarcity of land-fill space and due to its ever increasing cost, waste materials and by-products utilization has become an attractive solution to environmental and ecological problems. In fact, animal and agroindustrial by-products are protein-rich materials, as a very important bioresources that could be utilized for applications in food, health-care products, and pharmaceuticals [2–5].

Proteins could be modified by treatment with proteolytic enzymes, thus, the functional properties and nutrition value of proteins could be improved by enzymatic hydrolysis [6], which can generate polypeptides and free amino acids [7,8]. Otherwise, protein hydrolyzates can be used as emulsifying agents in a number of applications such as salad dressings, ice cream, coffee whitener, spreads, and emulsified meat products like sausages or luncheon meat. However, the enzymatic treatment of various food proteins often results in a bitter taste due to the formation of low molecular weight peptides composed mainly of hydrophobic amino acids [9]. Thus, the formation of bitter peptides was the most serious problem in the practical use of food protein hydrolyzates.

Aminopeptidases are exopeptidases that selectively release *N*-terminal amino acid residues from polypeptides and proteins. Moreover, these enzymes from various sources can be used successfully to debitter protein hydrolyzates. The leucine aminopeptidases (LAP, E.C. 3.4.11.1) are zinc-containing exopeptidases that belong to the M17 peptidase family [10]. The potential of LAPs has been reported in various industrial processes, including the preparation of debittered hydrolysates [11] and the conversion of l-homophenylalanyl amide into l-homophenylalanine, the versatile intermediate for a class of angiotensin-1-converting enzyme inhibitors [12]. LAP can also improve flavor development [13]. For these reasons, much attention has been paid on this enzyme. The leucine aminopeptidases are ubiquitous, especially in various microbial sources. Lin *et al*. have cloned and characterized a LAP from the *Bacillus kaustophilus* [14]. The importance of Thr-346 and Leu-352 residues in *Bacillus kaustophilus* leucine aminopeptidase (BkLAP) was explored by site-directed mutagenesis [15]. Further research indicated that residues Ala-348 and Gly-350 were essential for BkLAP in maintaining a stable active-site environment for the catalytic reaction [16]. Despite these, detailed information on how various factors (e.g., pH, temperature and divalent cations) on the activity and secondary structures of BkLAP is still unknown. Furthermore, the biological significance and the applied aspect of this enzyme are still not fully understood.

This topic promoted a number of theoretical studies on the environmental effects of divalent cations, temperature, pH in the activity and structure of the enzyme. In addition, the potential applications of BkLAP in the hydrolysis of fishery by-product (Anchovy) were also determined. The data presented here not only considerably expand our basic knowledge about this enzyme, but also provide the useful information for its application in food processing industry.

## 2. Results and Discussion

### 2.1. Expression and Purification of the Recombinant Enzyme

To obtain the optimal conditions for the production of active BkLAP, many efforts have been made in heterogeneous gene expression of BkLAP in *E. coli* BL21 (data not shown), including the IPTG concentration, induction temperature and time, and so on. Finally, IPTG at a final concentration of 0.05 mM, induction temperature and time of 28 °C and 8 h respectively were considered to be the best conditions and were further used in subsequent experiments. Under these conditions, the recombinant protein (BkLAP) showed active and soluble in the supernatant of the cell lysate.

**Figure 1:**
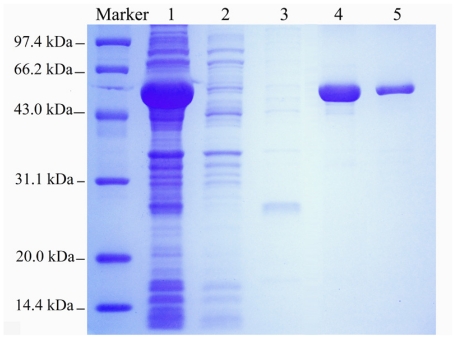
SDS-PAGE analysis of total cell proteins from *E. coli* BL21 (harboring pET28a-LAP) and the eluted fractions from nickel-chelate chromatography. Lane **1**: total cell lysate; Lane **2**: eluted with washing buffer containing 50 mM imidazole; Lane **3**: eluted with washing buffer containing 100 mM imidazole; Lane **4**: eluted with washing buffer containing 200 mM imidazole; Lane **5**: samples filtered through a Cross Flow Ultra-filtration Cassette with a molecular weight cutoff of 10,000 Da.

The BkLAP in the crude extract was further purified by nickel column chromatography and eluted with the washing buffer containing various concentrations of imidazole. As can be seen from the electropherogram, the 200 mM eluent contained most of the target protein (Figure 1). The enzymatic activity of the eluent fraction was further confirmed by enzyme assay. SDS-PAGE analysis of the purified proteins exhibited a predominant protein band with apparent molecular weight of approximately 54 kDa (Figure 1), in close agreement with the results of Lin *et al*. [14]. The pooled fractions were further filtered through a Cross Flow Ultra-filtration Cassette with a molecular weight cutoff of 10 kDa to remove the imidazole (Figure 1). Finally, the BkLAP was successfully expressed and purified for further testing.

### 2.2. Biochemical Properties and Conformation Changes of the BkLAP under Different Conditions

#### 2.2.1. Effect of pH on the Enzymatic Activity and Secondary Structure of BkLAP

The enzyme was active in a narrow pH ranged from 7.0 to 9.0, with optimum activity at pH 8.0, and coincidence with the results of Lin *et al*. [14]. However, in the same pH of 8.0, the enzymatic activity also exhibited variation between different buffers (50 mM McIlvanie buffer, 50 mM potassium-phosphate buffer, 50 mM HEPES buffer, 50 mM Tris-HCl buffer), with optimum activity at 50 mM potassium-phosphate buffer of pH 8.0 (Table 1). These results implied that some ions might interact with the active site of BkLAP, and thus inhibited the protease activity. The enzymatic activity was almost lost when the pH was lower than 6.0 or higher than 10.0 (Table 1).

Circular dichroism (CD) was a useful tool to probe the possible conformational transition and the changes in secondary and tertiary structures. As can be seen from the results, the main backbone conformation of BkLAP at pH of 8.0 was α-helix (27.67%), β-sheet (38.10%), turn (5.50%) and random (28.67%) (Table 2). However, the content of these structures changed when BkLAP dissolved in other buffer of pH value. These results indicated that the solution of different pH could influence the secondary structure of protein molecules and further resulted in enzymatic activity changes. Meanwhile, the different secondary structures all play an important role in the enzymatic function of BkLAP, with a little change could result in dramatic change on the enzymatic activity. Present results could partly explain why BkLAP had no enzymatic activity in lower pH value of 4.0 and 6.0. At pH 10, BkLAP had almost lost its enzymatic activity. However, the secondary structure of BkLAP at pH 10 was highly similar to that of pH 8 (Table 2). This result implied that the general acid and general base may also play an important role in the development of enzymatic activity of BkLAP. The detailed mechanism still needs further research.

#### 2.2.2. Effect of Temperature on the Enzymatic Activity and the Secondary Structure of BkLAP

**Figure 2:**
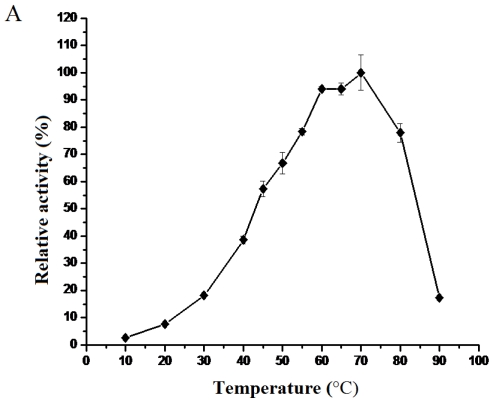

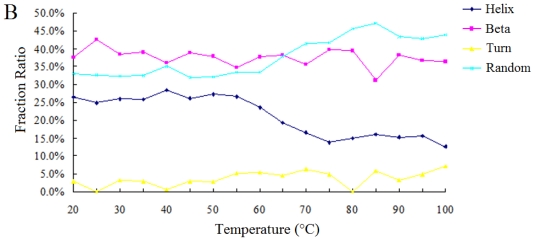
Effects of temperature on the enzymatic activity and secondary structures of the purified BkLAP. (**A**) Relative activity of BkLAP at different temperatures; (**B**) Variance of different secondary structures of BkLAP under different temperatures. For the detailed methods, please see the section 3.4 and 3.5.

The effect of temperature on the activity of BkLAP was determined in 50 mM potassium-phosphate buffer (pH 8.0). The enzymatic activity of the BkLAP increased with the raising of the temperatures. The optimum reaction temperature of the enzyme was at 70 °C, and was similar with the results of Lin *et al*. [14], which found the optimal temperature of the enzyme was 65 °C. However, the enzymatic activity declined quickly when the temperature was more than 70 °C, and increasing the temperature to 90 °C resulted in almost complete loss of enzymatic activity (Figure 2(A)).

In the present paper, the effect of temperature on the secondary structure of BkLAP was also tested to try to explain the mechanism from molecular structure point of view. The results of CD were shown in Figure 2(B). The α-helical content of BkLAP greatly decreased from approximately 27% to 15% with the increasing of the temperature. Meanwhile, the random structure increased from approximately 32% to 43% as the temperature rose, which means that the structure experienced the unfolding and degenerating process. The α-helix structure of BkLAP was relatively stable at temperatures in the range of 20 °C to 55 °C, however, the α-helix structure decreased gradually with the raising of the temperature. These results were in accordance with the present results of the enzymatic activity at different temperatures. When the temperature rose to 70 °C, the secondary structure of BkLAP was greatly changed, and caused the deactivation of the enzyme.

#### 2.2.3. Effect of Various Divalent Cations on the Enzymatic Activity and Secondary Structure of BkLAP

The effect of different chemicals on enzymatic activity was shown in Table 3. Among the divalent cations studied, Ni^2+^ ions have a strongly stimulatory effect on the catalytic activity of BkLAP (Table 3) and the next strongest effects were produced by Mn^2+^ and Co^2+^ ions. These results agreed with those of Lin *et al*. [14], and further indicated that the same LAP from *B. kaustophilus* CCRC 11223 was expressed. Metallo-aminopeptidases exhibit a broad range of metal-ion dependence and the LAP exhibited enhanced activity in the presence of several metal ions, like other cobalt-activated metalloenzymes [17,18]. However, enzymatic activity of BkLAP was strongly inhibited by Hg^2+^, with only 6% of activity left after treating with Hg^2+^ at the concentration of 1×10^−4^ M. Inhibition by Hg^2+^ may indicate the importance of indole amino acids in enzyme function [19], as has been demonstrated for the LAP of *Lactobacillus curvatus* DPC2024 [17]. Meanwhile, all other metal ions tested here all showed inhibitory effect on the enzymatic activity of BkLAP.

**Figure 3:**
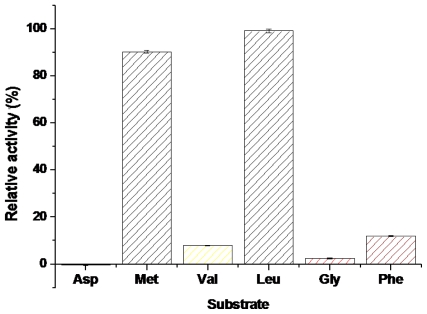
Substrate selectivity of the purified BkLAP. Enzymatic activities were measured with various substrates under standard assay conditions.

Different conformation of secondary structure was observed when various divalent cations were added to the solution. The secondary structure of BkLAP was slightly modified after Ni^2+^ was added (Figure 3). A small increase in α-helix structure (from 27.67% to 30.73%) and random structure (from 28.67% to 29.07%) were observed. Meanwhile, β-sheet and the turn structure slightly decreased from 38.10% to 36.47% and from 5.5% to 3.77%, respectively (Table 4). However, no statistical significance was found (*P* > 0.05). In addition, the secondary structure of BkLAP was greatly modified after Hg^2+^ was added to the buffer. Compared to the control group where no divalent cations were added, the significant decrease of β-sheet content (from 38.10% to 0.00%), and an increase of α-helix (from 27.67% to 37.07%), turn (from 5.5% to 28.77%) and random structure (from 28.67% to 34.17%) were observed after Hg^2+^ was added (Table 4). Hg^2+^ had an inhibitory effect on the enzymatic activity of BkLAP as previously mentioned. In the present results, Hg^2+^ caused great change on the secondary structure of the BkLAP, which may be the reason to induce the BkLAP completely lost its activity. The significant modification of β-sheet and turn structure indicated that they also played an important role on the LAP activity of the enzyme. Other divalent cations also have some effect on the secondary structure of the BkLAP, which could partly explain why the enzymatic activity of BkLAP changed (Table 4).

#### 2.2.4. The Substrate Selectivity of BkLAP

Among various *p*-nitroanilide derivatives tested here, the BkLAP was most active on Leu-*p*-NA (100%), followed by Met-*p*-NA (90%), meanwhile, the BkLAP also showed hydrolytic activity to Phe-*p*-NA (12%), Val-*p*-NA (8%) and Gly-*p*-NA (2%), and no activity was found to Asp-*p*-NA (Figure 3). Lin *et al*. found that the LAP was most active on Leu-*p*-NA (100%), followed by Cys-*p*-NA (9.7%), Ala-*p*-NA (1.7%), and Pro-*p*-NA (1.4%) [14], those results together with the present results showed that the enzyme has wide substrate selectivity.

### 2.3. BkLAP Greatly Promoted the Hydrolysis of Anchovy Protein

*Engraulis japonicus* anchovy is one of the main swimming fishes in China. They are found in extensive areas throughout the Yellow Sea and Bohai large reserves, with an estimated annual harvest of 700–800 metric tons [20]. They are usually processed as feedstuffs because of their easy decay and small body. Anchovy is rich in protein (up to 15–20%) and possess abundant of endogenous enzyme [21], so they can be used as raw material for production of protein hydrolysate with high added value.

It has been proved that hydrolysates of protein produced with single proteases often show a limited degree of hydrolysis. One enzyme cannot achieve such a high degree of hydrolysis (DH) in a reasonable period of time [22,23]. A higher degree of hydrolysis can be obtained with combinations of endo- and exo- protease containing preparations such as Alcalase and Flavourzyme [24]. Accordingly, four groups of experiments were designed in the present studies to evaluate the hydrolytic activity of BkLAP, and explore the possible combined protease recipe for the efficient production of protein hydrolysates of anchovy (Table 5).

**Figure 4:**
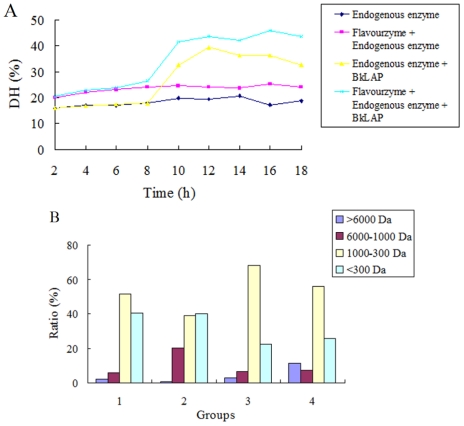
Degree of hydrolysis (DH) and the corresponding molecular weight distribution (%) of different experimental groups designed for the hydrolysis of anchovy proteins. (**A**) DH of different experimental groups; (**B**) Molecular weight distribution (%) of peptides in different hydrolysates groups. For detailed methods, please see the section 3.6.2, 3.6.3 and 3.6.5.

The DH of anchovy was determined and the results were shown in Figure 4(A). The DH of anchovy protein was about 16% after 6 h of autolysis by endogenous enzyme (group 1), and it increased to be approximately 24% when Flavourzyme was combined with endogenous enzyme (group 2). For groups 3 and 4, after 6 h of hydrolysis, the BkLAP protease was added, and it was found that the DH of anchovy protein increased quickly to 38% (group 3) and 45% (group 4). The results indicated that BkLAP protease has good hydrolytic activity, and BkLAP showed positive coordination in the hydrolysis of anchovy protein by comparing the DH value of group 3 with that of group 4.

The molecular weight distribution of hydrolysates (Figure 4(B)) showed that all the hydrolysates were rich in fractions with smaller molecular weight (less than 1,000 Da), thus corroborating well with the higher DH observed in the study and indicating that proteins were greatly degraded by protease. Quist *et al*. found that the proteins were degraded into high molecular weight peptides by endoproteases firstly, then the exoproteases treated sequentially to dissociate them into smaller fragments [8]. Flavourzyme was a mixture of enzyme that contains both exopeptidase activity and endopeptidase acticity. Accordingly, compared with the group 1, the molecular weight distribution of >6,000 Da was significantly decreased in group 2. This means that the Flavourzyme promoted the hydrolysis process. Moreover, the large molecular weight distribution of 6,000–1,000 Da was also significantly decreased when BkLAP was combined with Flavourzyme in group 4, compared with the corresponding group of 2. These results indicated that as an exopeptidase, the role that BkLAP plays in the hydrolysis of anchovy proteins can not be ignored. Moreover, BkLAP is a good candidate to be used to remove the bitterness in protein hydrolysis industry, because the peptide with molecular weight distribution of 6,000–1,000 Da in protein hydrolysate is often the cause of bitterness [9]. However, the large molecular weight distribution of >6,000 in group 4 may be caused by the variation of reaction temperature and pH after BkLAP was combined in the hydrolysis and further interfered with the enzymatic activity of Flavourzyme or endogenous enzyme. Therefore, further research should focus on the process optimization of hydrolytic conditions in the hydrolysis of protein substrates.

The amino acid composition of different hydrolysates was shown in Table 6. Positively coordinated proteolysis of anchovy, which was catalyzed by combination of endogenous enzyme of anchovy, Flavourzyme and BkLAP, seemed to be the most efficient in liberating amino acids and this was most noticeable in group 4 after 18 h of hydrolysis. BkLAP, as an aminopeptidase, played an important role in the hydrolysis to generate free amino acid, especially the leucine, arginine and lysine. Essential amino acids: His, Tyr, Val, Thr, Ile, Leu, Arg, Phe were observed in hydrolysates and all showed a high value, which indicated that the anchovy hydrolysates was rich in nutrients and the amino acid composition would fulfill human requirements. Therefore, anchovy as a quality protein source may have a wide application such as nutritional additives, flavor enhancers, functional ingredients, and so on.

## 3. Experimental Section

### 3.1. Total Gene Synthesis of Leucine Aminopeptidase and Construction of Expression Plasmid

The full length of the leucine aminopeptidase gene from *Bacillus kaustophilus* CCRC 11223 was acquired by the PCR-based two-step DNA synthesis method of Xiong *et al*. [25] with minor modifications. According to the gene sequence submitted to NCBI (GenBank: AY308074.1), 15 pairs of oligonucleotides (60–65 mer) were designed and synthesized. There was a 15 mer overlap for each of the oligonucleotides used, with the first and last oligonucleotides harboring a restriction endonucleases site of *BamHI* and *XhoI*, respectively. The gene was divided into three DNA fragments, and each fragment was synthesized by assembling five pairs of oligonucleotides by PCR. PCR reactions were carried out in one cycle at 94 °C for 2 min, followed by 25 cycles of 90 °C for 30 s, 45 °C for 45 s and 72 °C for 50 s, with 2.5 U *pfu* Taq polymerase. The final cycle was followed by an additional 10 min at 72 °C to ensure complete extension for all PCR reactions, unless stated otherwise. And then, the resulting PCR fragments were purified, mixed and used to assemble the template for the second PCR reaction by overlap extension PCR. The second PCR reaction was performed with the two outermost oligonucleotides as primers. The PCR reaction was carried out in one cycle at 94 °C for 2 min (during which 2.5 U *pfu* Taq polymerase were added), then 25 cycles of denaturing at 94 °C for 30 s, annealing at 60 °C for 35 s, extension at 72 °C for 2 min. The PCR amplified products were purified with 1% agarose gel electrophoresis. The object gene was cloned into cloning vector pBluescript SK (Stratagene, La Jolla, CA, USA) and was used to transform *E. coli* DH5α (Takara, Dalian, China). The recombinant plasmids were extracted using plasmid mini kit (TaKaRa, Dalian, China) and sequenced to ensure its sequence accuracy. The plasmids with the right sequence were digested with the restriction endonucleases and linked to expression vector pET28a (Stratagene, La Jolla, CA) to form pET28a-LAP. The recombinant plasmid was used to transform *E. coli* BL21 (Takara, Dalian, China).

### 3.2. Gene Expression and Enzyme Purification

For expression of recombinant leucine aminopeptidase (BkLAP), *E. coli* BL21 cells harboring pET28a-LAP were grown at 37 °C in 1.0 L of LB medium containing 2 mL of kanamycin, and induced at an optical density of 0.8 at 600 nm by IPTG to a final concentration of 0.05 mM. After 8 h of induction at 28 °C, the cells were harvested, resuspended in 100 mL of 20 mM Tris-HCl (pH 8.0) and disrupted by sonication (ULTRASONIC PROCESSOR UH-950A, Tianjin Autoscience instrument Co., Ltd., Tianjin, China). The cell lysates were then centrifuged at 11,000 g for 20 min to remove the insoluble cell debris, and the supernatants were assayed for further purification.

For further purification of BkLAP, the supernatant was then filtered through a 0.45 μm filter and applied to a Ni^2+^-NTA-agarose column (bed volume 40 mL). Ni^2+^-nitrilotriacetate (Ni^2+^-NTA) resin was obtained from Qiagen Inc. (Valencia, CA, USA). The column was washed with washing buffer (20 mM Tris-HCl, pH 8.0) until the eluant had an A280 of less than 0.01, followed by 50 mM and 100 mM imidazole added to the washing buffer, and finally the enzyme was eluted with 200 mM imidazole. The enzyme containing eluent was filtered through a Cross Flow Ultra-filtration Cassette with a molecular weight cutoff of 10,000 Da to remove the imidazole while simultaneously adding into the potassium-phosphate (pH 8.0) buffer and concentrated to a certain volume.

### 3.3. Electrophoresis and Protein Assay

The purified enzyme was run on a 10% SDS-PAGE gel according to Laemmli [26]. Before electrophoresis, *E. coli* cells collected from 1 mL of culture broth or the purified protein were mixed with 5 × loading buffer, heated at 100 °C for 5 min, and centrifuged at 14,000 g for 5 min. The proteins were visualized by staining with Coomassie Brilliant Blue R-250.

### 3.4. Enzyme Assay

BkLAP activity was assayed by monitoring the hydrolysis of l-leucine-*p*-nitroanilide (l-Leu-*p*-NA) according to the method of Kuo *et al*. [27] with a little modification. The reaction mixture contained potassium-phosphate buffer (pH 8.0), 1 μL of 0.01 M Ni^2+^, an appropriate amount of the purified enzyme to a total volume of 100 μL. The mixture was preheated at 70 °C for 3 min, and then 10 μL of 20 mM Leu-*p*-NA was added and incubated at the same temperature for 10 min longer. 100 μL of absolute alcohol was added to terminate the reaction. The absorbance at 405 nm was then measured. One unit (U) of LAP activity is defined as the amount of enzyme releasing 1 μmol of *p*-nitroanilide per minute under the assay conditions. The hydrolytic activity of the purified enzyme against several other *p*-nitroanilide derivatives was also determined according to the above method.

For the effect of pH and various buffer on BkLAP activity, the assays were performed at 70 °C in 50 mM citrate-phosphate buffer (pH 3–7), 50 mM McIlvanie buffer (pH 3–8), 50 mM potassium-phosphate buffer (pH 6–8), 50 mM HEPES buffer (pH 7–8) 50 mM Tris-HCl buffer (pH 8–9) and 50 mM Gly-NaOH buffer (pH 9–12) and assayed under above conditions. For the effect of temperature on BkLAP, activity assays were performed in 50 mM potassium-phosphate buffer (pH 8.0) at different temperatures ranging from 10 °C to 90 °C.

### 3.5. Circular Dichroism Spectroscopy

CD measurements were carried out with a JASCO J-815 spectropolarimeter (Jasco, Tokyo, Japan) equipped with a cell holder thermostatically controlled by circulating water from a bath. The instrument was controlled by Jasco’s Spectra Manager^TM^ software. All the measurements were performed under nitrogen flow. The spectra were recorded over a wavelength range of 190–250 nm using a cuvette of 1 mm and at a scan speed of 100 nm/min and a response time of 1 second. Photomultiplier absorbance did not exceed 600 V in the analyzed spectral region. An appropriate buffer solution run under the same conditions was taken as a blank and subtracted from the sample spectra. Each spectrum was the average of three scans and detected at room temperature. Protein concentration (0.25 mg/mL) that dissolved in different pH buffer (4.0, 6.0, 8.0, 10.0) or metal ions were tested. All the CD spectra were analyzed by the Yang method with the manager software. Thermal denaturation experiments were performed by monitoring the ellipticity at 222 nm. The temperature was increased with a heating rate of 2 °C per minute from 20 °C to 100 °C.

### 3.6. Anchovy Hydrolytic Experiment

#### 3.6.1. Materials

Anchovy (*Engraulis japonicus*) were purchased from Zhoushan supermarket in Zhejiang, China. Fresh fish after capture were collected and stored in a polyethylene bag at −80 °C until used. Fish was ground to uniformity before used for the present experiments. Flavourzyme 500 MG was obtained from Novozymes China Inc. (Jinan, Shandong, China).

#### 3.6.2. Production of Protein Hydrolysate with Commercial Enzymes Combined with BkLAP

Due to the mind of anchovy protein has the endogenous enzymatic hydrolysis activities, four groups were designed to comparison the combination effect of BkLAP with other enzyme on the hydrolysis of anchovy protein. The combination of the enzymes for the four groups was designed as follows: In the first group, using endogenous enzyme only. In the second group, using Flavourzyme enzymes combined with the endogenous enzyme. In the third group, using BkLAP combined with the endogenous enzyme. In the fourth group, using BkLAP combined with the endogenous enzyme and Flavourzyme enzymes. In the last two groups, two steps of hydrolysis were designed: For the third group, the protein was first hydrolyzed with endogenous enzyme for 6 h, and then BkLAP was added for further hydrolysis at its optimal conditions. For the fourth group, the protein was first hydrolyzed with the endogenous enzyme and Flavourzyme enzymes for 6 h, and then BkLAP was added for further hydrolysis at its optimal conditions. Parameters for enzymatic hydrolysis of anchovy protein were shown in Table 5. The hydrolysis experiments were carried out using the pH-stat method in a 250 mL glass reactor under controlled conditions. During hydrolysis, pH and temperature was maintained at the optimal value of the enzyme respectively by addition of 2 N NaOH. A 25 g portion of mind was suspended in 75 mL of deionized water and enzyme was added to a final concentration of 2,000 U of Flavourzyme and 50 U of BkLAP respectively. The reaction was in thermostatic water bath with stirring speed of 200 rpm/min. The time of hydrolysis for the experimental design was 18 h and samples were got with 2 h intervals. Reactions were terminated by heating the mixture in boiling water for 20 min. The hydrolysate was then centrifuged at 10,000 g for 30 min and the supernatant was filtrated using a microfilter of 0.22 μm in order to remove suspended solids. The supernatant was collected and frozen at −70 °C for further testing.

#### 3.6.3. Measurement of Degree of Hydrolysis (DH)

DH was defined as the percentage of free amino groups cleaved from proteins, which was calculated from the ratio of α-amino nitrogen to total nitrogen. According to the method of Nilsang *et al*. [28], the amino nitrogen content was determined by a formaldehyde titration method. The total nitrogen content was determined by Kjeldahl method (the conversion factor was 6.25 for loach protein) [29].

#### 3.6.4. Free Amino Acid Analysis

Free amino acid composition was determined by reversed-phase HPLC analysis of 6-aminoquinolyl-*N*-hydroxysuccinimidyl carbamate (AQC) derivatives using g-aminobutyric acid as internal standard. The amino acids were treated with AQC to form AQC derivatives, which were then analyzed using a Waters HPLC system (Millipore Ltd., Watford, UK) fitted with a reversed-phase C_18_ column.

#### 3.6.5. Molecular Weight Distribution and Free Amino Acids Contents Anlaysis

The molecular weight distributions of different peptides in the hydrolysates were determined using high-performance size-exclusion chromatography (HP-SEC) on a Superdex Peptide HR 10/300 GL column (10 × 300 mm, Amersham Biosciences Co., Piscataway, NJ, USA) with a UV detector at 214 and 280 nm. The mobile phase (isocratic elution) was 0.02 M sodium phosphate buffer containing 0.25 M NaCl (pH 7.2), at a flow rate of 0.5 mL/min. A molecular-weight calibration curve was prepared from the average elution volume of the following standards: cytochrome C (12,500 Da), aprotinin (6,500 Da), vitamin B12 (1,355 Da), Gly-Gly-Gly (189 Da) and Glycine (75 Da) (Sigma Co., Saint Louis, MO, USA). UNICORN 5.2 software (Amersham Biosciences Co., Piscataway, NJ, USA) was used to analyze the chromatographic data.

### 3.7. Statistical Analysis

All experiments were performed in triplicate. The separation of means was accomplished using one-way analysis of variance (ANOVA) (*P* < 0.05) with the aid of SPSS 13.0 for Windows software. All the curves were fitted with Microsoft Office Excel 2003.

## 4. Conclusions

The secondary structure of BkLAP had close association with its enzymatic function. Environmental factors could induce the conformational alteration of BkLAP and thus influence its activity. BkLAP showed great potential to be used in protein hydrolysis industry. Through combination with other commercial enzymes, such as Flavourzyme, the BkLAP possess highly potential to hydrolyze anchovy protein to increase the free amino acid contents. Further research will focus on the process optimization of hydrolytic conditions in the hydrolysis of various protein substrates, especially the agricultural by-product, to produce valuable products.

## Figures and Tables

**Table 1:** Effect of various pH and buffers on the enzymatic activity of the purified *Bacillus kaustophilus* CCRC 11223 (BkLAP).

pH	Citrate-phosphate buffer	McIlvanie buffer	HEPES buffer	Potassium-phosphate buffer	Tris-HCl buffer	Glycine-NaOH buffer
3.0	0.00 ± 0.00	0.06 ± 0.00	—	—	—	—
4.0	0.00 ± 0.00	0.10 ± 0.02	—	—	—	—
5.0	0.14 ± 0.07	0.49 ± 0.18	—	—	—	—
6.0	0.66 ± 0.08	0.75 ± 0.08	—	4.43 ± 0.56	—	—
7.0	0.31 ± 0.06	2.81 ± 2.79	44.70 ± 3.32	63.59 ± 3.05	—	—
7.5	—	12.38 ± 1.29	53.10 ± 2.15	—	—	—
8.0	—	56.26 ± 7.42	31.44 ± 6.67	100.00 ± 0.58	40.45 ± 0.94	—
9.0	—	—	—	—	39.32 ± 0.99	1.79 ± 1.63
10.0	—	—	—	—	—	1.86 ± 1.61
11.0	—	—	—	—	—	2.19 ± 1.65
12.0	—	—	—	—	—	0.00 ± 0.00

Note: Enzymatic activities were measured at various pH of different buffers under standard assay conditions. Number in the table shows the relative activity of the enzyme compared to the value in potassium-phosphate buffer of pH 8.0 (as 100). “—” represents not tested.

**Table 2:** Fractions of different secondary structures of BkLAP that dissolved in various pH buffers.

	pH 4.0	pH 6.0	pH 8.0	pH 10.0
Helix	43.87% a	32.40% b	27.67% c	25.57% c
Sheet	0.00% a	37.27% b	38.10% b	40.27% b
Turn	32.83% a	1.53% b	5.50% b	7.30% b
Random	23.27% a	28.77% b	28.67% b	26.83% b

a–c Note: Values with different superscripts in the same line were significantly different at level of 0.05.

**Table 3:** Effect of various divalent cations on the enzymatic activity of the purified BkLAP.

Metal ions	Concentration (M)	Relative activity (%)
None	—	100
Hg^2+^	1×10^−4^	6
Mg^2+^	1×10^−4^	39
Fe^2+^	1×10^−4^	99
Fe^3+^	1×10^−4^	48
Ca^2+^	1×10^−4^	32
Ba^2+^	1×10^−4^	101
Cu^2+^	1×10^−4^	48
Zn^2+^	1×10^−4^	22
Li^2+^	1×10^−4^	97
Co^2+^	1×10^−5^	141
	5×10^−5^	175
	1×10^−4^	202
	5×10^−4^	243
Mn^2+^	1×10^−5^	100
	5×10^−5^	420
	1×10^−4^	491
	5×10^−4^	755
Ni^2+^	1×10^−6^	380
	1×10^−5^	880
	5×10^−5^	1,007
	1×10^−4^	1,722
	5×10^−4^	3,500

Note: BkLAP was treated with potassium-phosphate buffer (pH 8.0) containing different concentrations of metal ions and enzymatic activities were measured under standard assay conditions. Enzymatic activity of none metal added was 100%.

**Table 4:** Changes in the different secondary structures of BkLAP treated with various divalent cations.

	None	Co^2+^	Ni^2+^	Zn^2+^	Cu^2+^	Mn^2+^	Hg^2+^	Fe^3+^	Ca^2+^
Helix	27.67% a	29.40% a	30.73% a	28.93% a	30.33% a	29.87% a	37.07% b	31.97% a	28.23% a
Sheet	38.10% a	35.83% a	36.47% a	36.10% a	35.23% a	36.83% a	0.00% b	32.50% a	37.77% a
Turn	5.50% a	6.20% a	3.77% a	5.87% a	6.10% a	4.43% a	28.77% b	6.67% a	6.47% a
Random	28.67% a	28.60% a	29.07% a	29.07% a	28.37% a	28.83% a	34.17% b	28.87% a	27.53% a

Note: The concentration of various divalent cations was 1 × 10^−4^ M. Enzymatic activities were measured under standard assay conditions.

a,b Values with different superscripts in the same line are significantly different at level of 0.05.

**Table 5:** Experiment design and parameters for enzymatic hydrolysis of anchovy protein.

Groups	Enzyme	Temperature (°C)	pH
1	Endogenous enzyme	55.0	7.0
2	Flavourzyme + Endogenous enzyme	50.0	6.5
3	Endogenous enzyme + BkLAP	55.0 °C for 6 h and then 70.0 °C for 12 h	7.0 for 6 h and then 8.0
4	Flavourzyme + Endogenous enzyme + BkLAP	50.0 °C for 6 h and then 70.0 °C for 12 h	6.5 for 6 h and then 8.0

**Table 6:** Free amino acid composition (g/100 mL) in anchovy protein hydrolysate of different experimental groups.

	Group 1	Group 2	Group 3	Group 4
Asp	139.03	179.62	249.74	305.93
Glu	181.22	203.05	387.26	461.61
Ser	135.09	225.65	159.20	294.60
Gly	60.06	90.83	120.17	203.97
His	115.55	235.00	149.35	253.23
Arg	347.31	398.17	434.78	541.84
Thr	95.54	182.94	168.62	241.27
Ala	173.87	230.44	283.05	379.32
Pro	27.48	45.99	50.40	75.08
Tyr	85.78	104.84	782.47	887.89
Val	138.53	211.30	221.81	324.37
Met	95.27	129.20	125.29	169.00
Cys	5.88	15.40	39.56	58.87
Ile	131.57	197.37	198.73	285.70
Leu	268.26	372.34	388.64	530.67
Phe	137.05	174.54	187.47	268.58
Lys	300.50	362.33	447.30	541.61
Total	2,437.99	3,359.03	4,393.82	5,823.54
